# Identification and Expression Analysis of CCCH Zinc Finger Proteins in Mulberry (*Morus alba*)

**DOI:** 10.3390/ijms26199490

**Published:** 2025-09-28

**Authors:** Feng Chen, Jie Yu, Zhi-Hong Han, Yong-Jin Deng

**Affiliations:** Sericultural Research Institute, Anhui Academy of Agricultural Sciences, Hefei 230000, China; chenfeng@aaas.org.cn (F.C.); agnesyu121@aaas.org.cn (J.Y.); hanzhihong@aaas.org.cn (Z.-H.H.)

**Keywords:** mulberry, CCCH, tissue expression analysis, qRT-PCR, *MaC3H33*

## Abstract

CCCH zinc finger proteins play critical roles in plant growth, development and stress responses. Here, 56 CCCH genes were identified in *Morus alba*. These genes displayed wide variation in coding sequence (456–6318 bp) and protein length (151–2105 aa), with most proteins predicted to localize in the nucleus and a few in chloroplasts, the endoplasmic reticulum or cytoplasm. Chromosomal mapping showed uneven distribution across 14 chromosomes, with tandem clusters on chromosomes 1, 6 and 13. Phylogenetic analysis classified 53 *MaC3Hs* into 13 subfamilies, while three genes remained ungrouped. Synteny analysis revealed four segmental duplication events, suggesting segmental duplication as the major expansion mechanism, under purifying selection. Comparative collinearity showed higher conservation with *Arabidopsis thaliana* than with rice or maize. Promoter analysis identified 22 cis-acting elements, mainly related to phytohormones, followed by abiotic stress and developmental regulation. Expression profiling under drought stress revealed differential expression across tissues, with *MaC3H33* showing strong induction (>200-fold in stems on day 6). Subcellular localization confirmed *MaC3H33* is nuclear, and yeast assays indicated no self-activation. These findings provide comprehensive insights into the *MaC3H* gene family and lay a foundation for functional studies related to drought tolerance in mulberry.

## 1. Introduction

As one of the most abundant and versatile families of transcriptional regulators in eukaryotes, zinc finger proteins are defined by their nucleic acid-binding capability, which allows them to control various processes of gene expression [1]. Among these proteins, the CCCH zinc finger proteins, identified by their distinctive cysteine cysteine cysteine histidine (CCCH) motif, represent a notable subgroup due to their involvement in RNA metabolism and post-transcriptional regulatory processes [2]. These proteins are associated with various key biological functions, including DNA protein and RNA protein [3]. Their significant regulatory roles underscore their importance in plant growth, developmental processes [1] and particularly stress response mechanisms [4].

Extensive research in plants has revealed that CCCH zinc finger proteins play essential roles in diverse developmental and physiological processes. They are known to regulate key stages of plant growth, including seed germination [5,6], flowering [7] and secondary wall synthesis [8], reflecting their broad and critical involvement in plant biology. For example, in *Arabidopsis*, *AtC3H59* influences multiple processes, including seed germination, seedling growth and seed development [9]. In wintersweet (*Chimonanthus praecox*), the *CpC3H3* gene promotes flowering and enhances the plant’s drought resistance [10,11]. In addition to their roles in development, CCCH proteins have attracted considerable interest for their ability to regulate plant responses to abiotic stresses, including drought [12], salinity [13,14], low temperature [15] and oxidative damage [16], thereby highlighting their essential contribution to plant resilience and survival under adverse environmental conditions. For example, *OsTZF5* is a key regulator that enhances rice tolerance to drought and cold stress [10,17]. In *Arabidopsis*, *AtC3H3* modulates salt stress responses by regulating the expression of salt tolerance related genes [18].

CCCH zinc finger proteins are particularly important in mediating drought responses, a critical consideration given that water deficit remains one of the most severe environmental stresses affecting plant development, yield and geographic range worldwide [19]. To survive drought stress, plants employ intricate adaptive mechanisms at the molecular, biochemical and physiological levels, in which both transcriptional and post-transcriptional regulators are key contributors [20]. Previous studies conducted on various plants such as rice [21], alfalfa *(medicago sativa* L.) [22] and poplar (*Populus ussuriensis*) [23] have illustrated the diverse roles of CCCH zinc finger proteins in regulating gene expression in response to drought stress, thereby modulating plant resilience and adaptation to water deficit conditions. For example, overexpressing *MsC3H29* in alfalfa significantly enhances primary root elongation and fresh biomass of transgenic hairy roots, and promotes drought resistance of alfalfa hairy roots by reducing ROS accumulation [22]. In rice, *OsTZF7* contributes to drought adaptation by regulating gene expression through ABA signaling pathways [24].

Despite the considerable advances in understanding the functional and mechanistic roles of CCCH in model species, the diversity, evolutionary relationship and precise functions of these proteins in non-model and economically important plants remain comparatively underexplored [10,18]. Mulberry *(Morus alba* L.) is one such economically significant plant that has attracted attention due to its ecological, agricultural and commercial importance [25]. Mulberry leaves serve as the primary food source for the silkworm (*Bombyx mori*), which underpins the silk industry. Additionally, the plant exhibits pharmaceutical properties with potential benefits for human health [26]. Furthermore, mulberry is known for its resilience to various environmental stresses, including drought, highlighting the importance of understanding the molecular basis of their stress tolerance mechanisms [27].

Currently, there is a lack of comprehensive genomic and functional research on CCCH in mulberry, and their specific contributions to plant growth, developmental processes, and stress tolerance remain largely unexplored. Therefore, identifying and characterizing the CCCH genes in mulberry is crucial for understanding their potential contributions to mulberry biology and stress tolerance mechanisms. Such information could ultimately facilitate genetic improvement strategies aiming to enhance drought tolerance and overall resilience in mulberry and related plant species. In this context, the present study aimed to systematically identify and characterize the CCCH zinc finger gene family in mulberry. A total of 56 *MaC3H* genes were identified in the genome of mulberry. We conducted comprehensive analyses of these genes, including structural features, phylogenetic relationships, promoter cis-elements, chromosomal distribution, microsynteny, subcellular localization and transcriptional activity. Considering the well-documented involvement of CCCH proteins in drought stress regulation in other plant species, we next analyzed the expression profiles of selected *MaC3H* genes in mulberry seedlings subjected to drought treatment. Using quantitative real-time PCR (qRT-PCR), we profiled their dynamic expression across different tissues (roots, stems and leaves) and at multiple time points during drought treatment. To better elucidate the functional role of MaC3H proteins, a gene *MaC3H33* that exhibits significant induction under drought stress was selected for further molecular characterization, exploring its subcellular localization and transcriptional activity. By combining genomic analyses, gene expression profiling and detailed molecular characterization, this study offers a comprehensive understanding of the potential roles of *MaC3H* genes in enhancing mulberry’s adaptability to environmental stresses, offering valuable foundations for future functional genomic studies and genetic improvement efforts in mulberry and beyond.

## 2. Results

### 2.1. Identification of the MaC3H Genes

Using BLASTP program identification, as well as Pfam and SMART verification, we identified 56 members of the *MaC3H* gene family *(MaC3H1*–*MaC3H56*). Detailed characteristics of these mulberry CCCH genes are provided in Table S1, including coding sequence (CDS) length, protein size, isoelectric point (pI), molecular weight (MW) and predicted subcellular localization. The CDS length varied from 456 bp in *MaC3H40* to 6318 bp in *MaC3H34*, corresponding to protein lengths ranging between 151 and 2105 amino acids. The calculated molecular weights extended from 17,106.22 Da in *MaC3H40* to 232,685.74 Da in *MaC3H34*. The theoretical pI values spanned from 4.73 in *MaC3H54* to 9.68 in *MaC3H15*. Subcellular localization prediction indicated that 39 proteins were exclusively nuclear, seven were distributed between the nucleus and chloroplast, four were targeted to mitochondria, one localized to the cytoplasm, and another was present in both the endoplasmic reticulum and nucleus.

**Figure 1:**
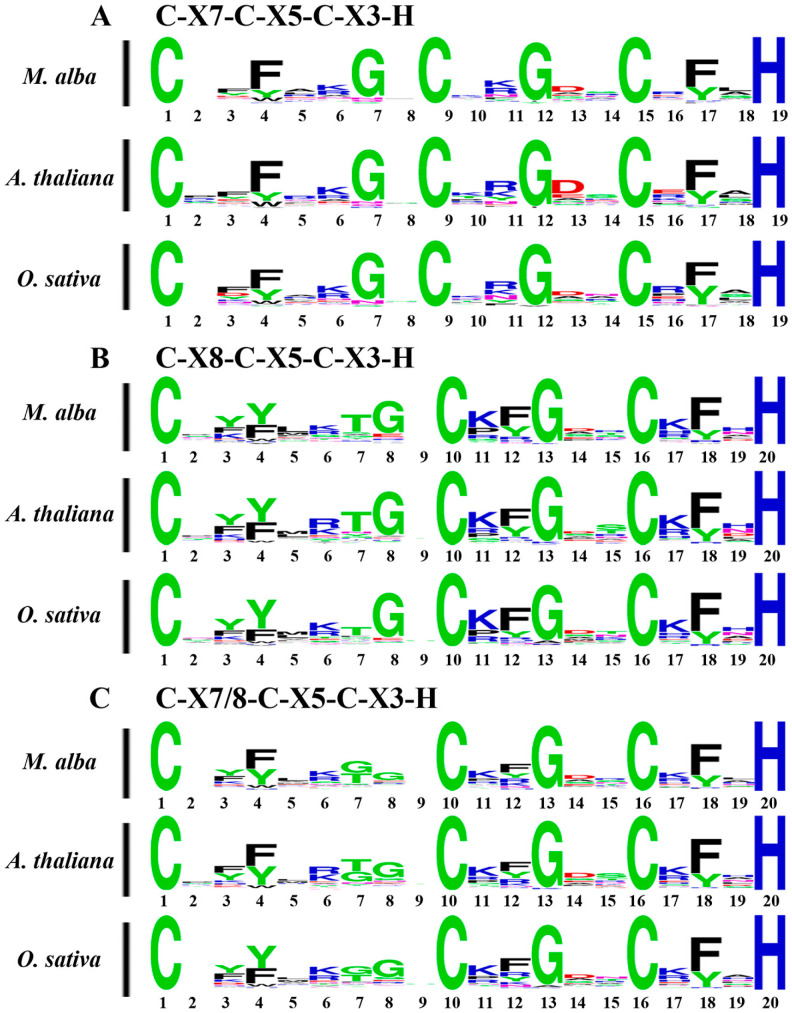
Sequence logos for common CCCH zinc finger motifs. (**A**) C-X_7_-C-X_5_-C-X_3_-H motifs of *M. alba*, *A. thaliana* and *O. sativa*. (**B**) C-X_8_-C-X_5_-C-X_3_-H motifs of *M. alba*, *A. thaliana* and *O. sativa*. (**C**) C-X_7_-C-X_5_-C-X_3_-H and C-X_8_-C-X_5_-C-X_3_-H motifs of *M. alba*, *A. thaliana* and *O. sativa*. The conservativeness of amino acids in different positions is indicated by the size of the characters.

A total of 57 sequences containing the C-X8-C-X5-C-X3-H motif and 43 sequences containing the C-X7-C-X5-C-X3-H motif were analyzed separately, and sequence logos were generated for each group (Figure 1). To investigate the structural features of CCCH motifs in MaC3H proteins, these motifs were compared with their homologs in *A. thaliana* and *O. sativa*. Composite sequence logos for the two motifs were also constructed, which clearly demonstrated a high level of conservation. Across all three motif types, four amino acid residues were found to be absolutely conserved, consistent with the motif profiles reported in the Pfam and SMART databases. Nevertheless, certain differences in motif characteristics were noted across the three plant species. For instance, in the *M. alba* C-X7-C-X5-C-X3-H motif logo, lysine was more frequently present at the C1+11 position than arginine, whereas the opposite pattern was observed in *A. thaliana* and *O. sativa*.

**Figure 2:**
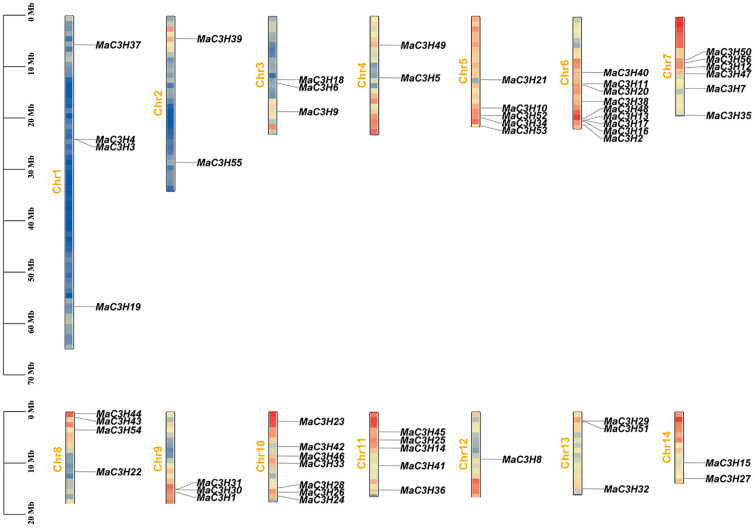
Chromosomal distribution of *MaC3H* gene family in *mulberry*. The scale on the left is in mega-bases. The gene names on chromosomes of right side represent the approximate locations of each *MaC3H* genes.

The chromosomal localization of *MaC3Hs* was analyzed, revealing an uneven distribution across the 14 chromosomes of mulberry (Figure 2). Chromosome 6 harbored the largest number of *MaC3Hs* (9), whereas chromosome 12 contained only one. Notably, many *MaC3Hs* were arranged in tandem clusters on the chromosomes. For instance, tandem clusters composed of two *MaC3Hs* were identified on chromosomes 1, 6 and 13.

### 2.2. Phylogenetic Relationships

**Figure 3:**
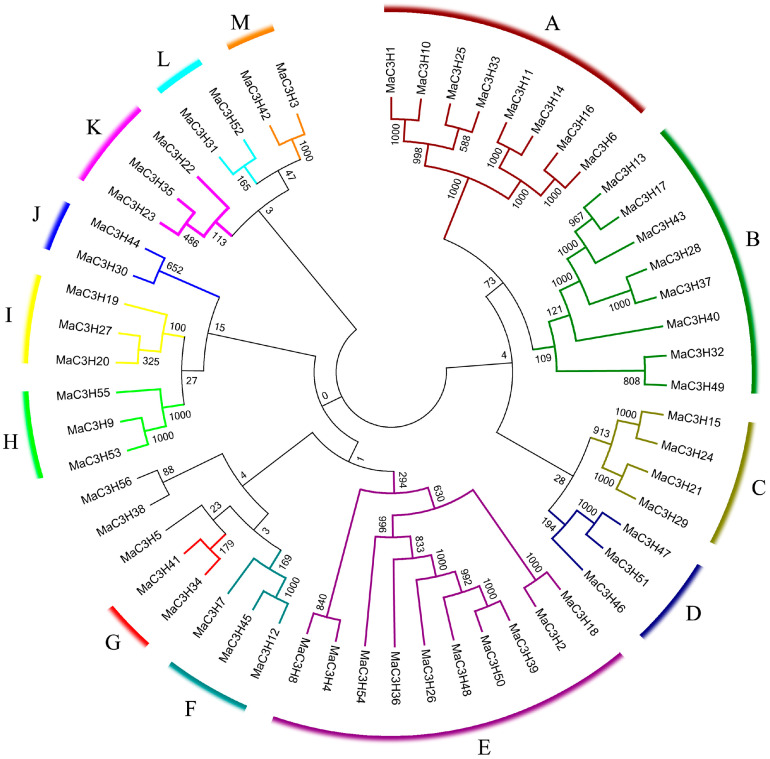
Phylogenetic analysis of CCCH in *mulberry*. 56 *MaC3H* genes are divided into 13 subfamilies (A–M).

To investigate the evolutionary relationships of mulberry CCCH-type zinc finger proteins, an unrooted phylogenetic tree was generated using the 56 predicted protein sequences, with 1000 bootstrap replicates providing statistical support (Figure 3) [28]. On the basis of bootstrap values greater than 100, these proteins were clustered into 13 subfamilies, designated CCCH (A-M). However, the *MaC3H5*, *MaC3H38* and *MaC3H56* genes were not assigned to any of these subfamilies, as their bootstrap support with other genes was below 100, a phenomenon also observed in CCCH genes of other plant species [29]. Among the defined groups, subfamily E represented the largest clade, containing 10 members. Subfamilies A and B each consisted of 8 proteins, while subfamily C included 4. Subfamilies D, F, H, I and K harbored 3 members apiece, whereas the remaining subfamilies (G, J, L and M) contained only 2 proteins each.

### 2.3. Motif and Gene Structure Analysis of MaC3Hs

**Figure 4:**
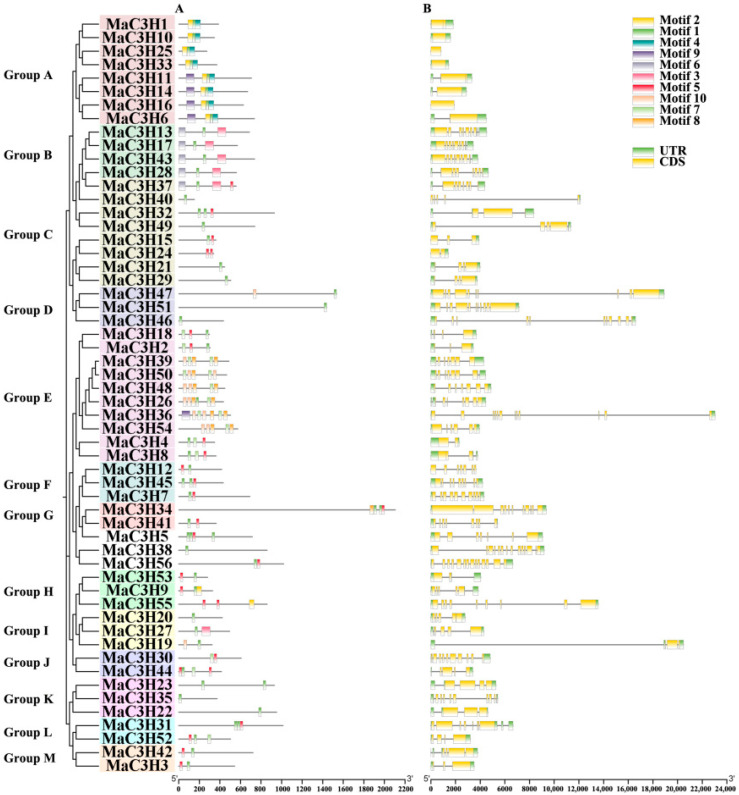
Conserved motifs and gene structure analysis of the mulberry CCCH gene family. (**A**) The motif composition of MaC3H proteins. The boxes with different letters and colors represent different sequences, and details of each conserved motif are in Table S2. (**B**) Exon and intron structures of the *mulberry* CCCH genes. Exons are indicated by yellow rectangles and introns by grey lines.

To gain deeper insight into the characteristics of *MaC3Hs*, we analyzed the motif composition as well as the intron–exon organization of each gene in this family. The presence of conserved motifs indicated that all *MaC3H* proteins contained at least one of motifs 1, 2, 4, 5, 7, 8 or 10, which correspond to the CCCH zinc finger domains, thereby confirming the accuracy of the identification of *MaC3H* genes in mulberry. The number and type of motifs were highly similar across the 13 subfamilies, although each subfamily exhibited distinct structural features (Figure 4A, Table S2). Members of the A subfamily consistently contained motifs 1, 2 and 4, and, except for *MaC3H36*, motif 9 was predominantly present in this group. Motif 6 was exclusively detected in five genes (*MaC3H13*, *MaC3H17*, *MaC3H28*, *MaC3H37* and *MaC3H43*) of the B subfamily. Genes belonging to the M subfamily possessed an identical number and type of motifs, which were located at conserved positions within the genes. Analysis of the intron–exon structures of the *MaC3H* genes revealed that only five genes (*MaC3H1*, *MaC3H10*, *MaC3H16*, *MaC3H25* and *MaC3H33*) in the A subfamily lacked introns, whereas the remaining genes contained between 1 and 13 introns (Figure 4B). Within each subfamily, the intron–exon organization was relatively conserved, with similar or even identical numbers of introns observed among members.

### 2.4. Synteny Analysis of MaC3Hs

**Figure 5:**
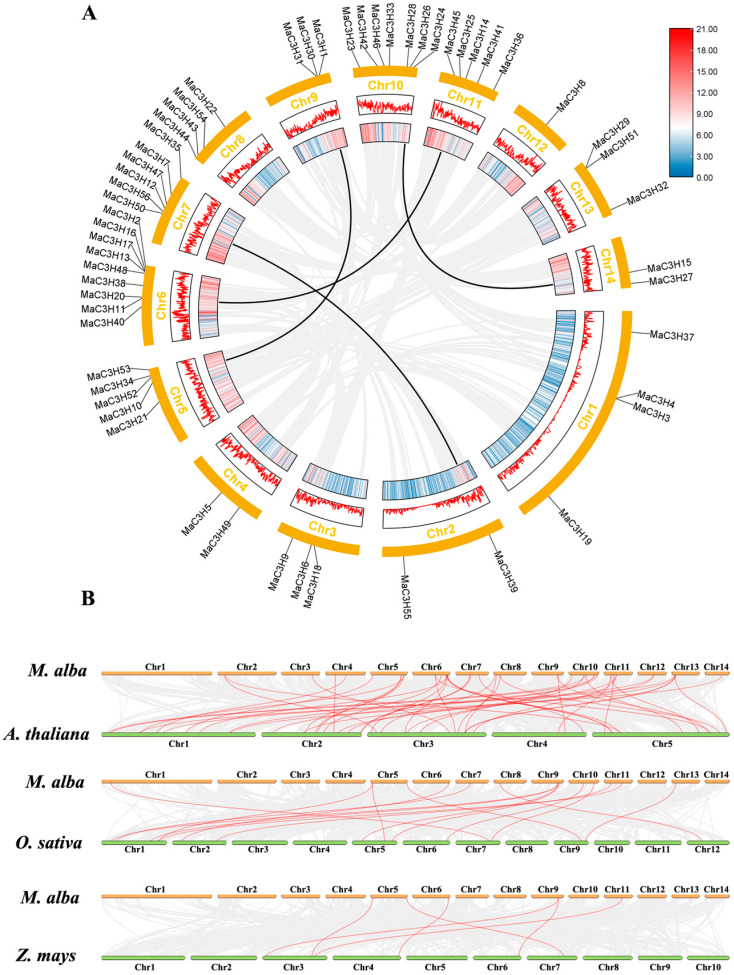
Synteny analysis of *MaC3H* genes. (**A**) Synteny analysis of *MaC3Hs* in *M. alba*. The gray lines represent all syntenic blocks in the genome of *M. alba*, and the black lines represent duplicate *MaC3H* gene pairs. (**B**) Synteny analysis of CCCH genes among *M. alba*, *A. thaliana*, *O. sativa* and *Z. mays*. The chromosomes of *M. alba* are represented in yellow, while the chromosomes of *A. thaliana*, *O. sativa* and *Z. mays* are represented in green. Gray lines represent the collinear blocks among *M. alba*, *A. thaliana*, *O. sativa* and *Z. mays*. Red lines indicate the orthologous relationships of CCCH genes among *M. alba*, *A. thaliana*, *O. sativa* and *Z. mays*.

Gene family expansion is typically driven by whole-genome duplication, segmental duplication, or tandem duplication events [30]. To investigate the potential genetic mechanisms underlying evolutionary processes, duplication events within the *MaC3H* gene family of *M. alba* were analyzed. Our analysis revealed four segmentally duplicated gene pairs involving eight *MaC3H* members, suggesting that segmental duplication has been the predominant force contributing to the diversification and expansion of the CCCH gene family in *M. alba* (Figure 5A, Table S3). To further evaluate the selective pressures acting on these duplicated genes, nonsynonymous (Ka) and synonymous (Ks) substitution rates were calculated with TBtools. As shown in Table S3, the calculated Ka/Ks ratios varied between 0.167 and 0.372, implying that purifying selection has been the major evolutionary constraint maintaining the functional stability of *MaC3H* genes in *M. alba*.

Additionally, synteny analyses were conducted among CCCH genes in *M. alba*, *A. thaliana*, *O. sativa* and *Z. mays* to further elucidate the phylogenetic relationships of *MaC3Hs*. As shown in Figure 5B (Table S4, Table S5 and Table S6), 45 homologous gene pairs were detected between *M. alba* and *A. thaliana*, while 13 pairs were identified between *M. alba* and *O. sativa* and only 5 pairs were observed between *M. alba* and *Z. mays*. The observed greater number of homologous gene pairs between *M. alba* and *A. thaliana* likely reflects their closer evolutionary relationship, in contrast to the fewer homologs identified with *O. sativa* and *Z. mays*.

### 2.5. Cis-Acting Element Analysis of MaC3Hs

**Figure 6:**
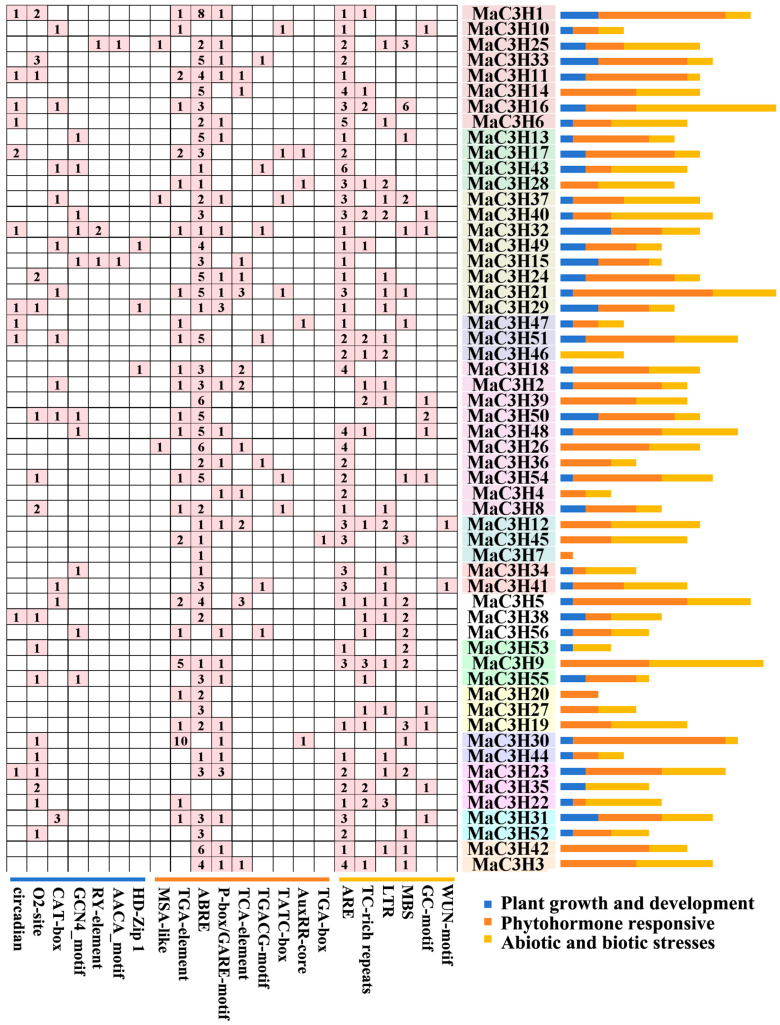
Analysis of cis-acting elements of *MaC3Hs*. Analysis of cis-acting elements in the promoter regions of *MaC3H* genes, numbers indicate the number of cis-acting elements, and histogram of cis-acting elements in each *MaC3H* genes.

Gene function is often regulated by cis-acting elements embedded within promoter sequences [31]. To gain deeper insights into the potential regulatory roles of *MaC3Hs* in mulberry, we retrieved 2000 bp upstream sequences from the start codon (ATG) of all 56 *MaC3H* genes and analyzed them using the PlantCARE database. A total of 22 types of cis-acting elements were identified and classified into three major categories: hormone-responsive elements, abiotic stress-responsive elements and elements related to plant growth and development (Figure 6, Table S7). Among these, hormone-responsive elements were the most abundant in the *MaC3H* promoter regions, including auxin-responsive, abscisic acid-responsive, gibberellin-responsive, jasmonic acid-responsive and salicylic acid-responsive elements. Except for *MaC3H35*, *MaC3H47* and *MaC3H54*, all promoters contained at least one hormone-responsive element. These findings suggest that *MaC3Hs* may participate in multiple plant hormone signaling pathways. Elements associated with abiotic stress responses—such as those responsive to wounding, low temperature, drought, anaerobic conditions and general defense or stress signals—were also identified, although their overall abundance was relatively low.

### 2.6. Functional Annotations of MaC3H Genes

**Figure 7:**
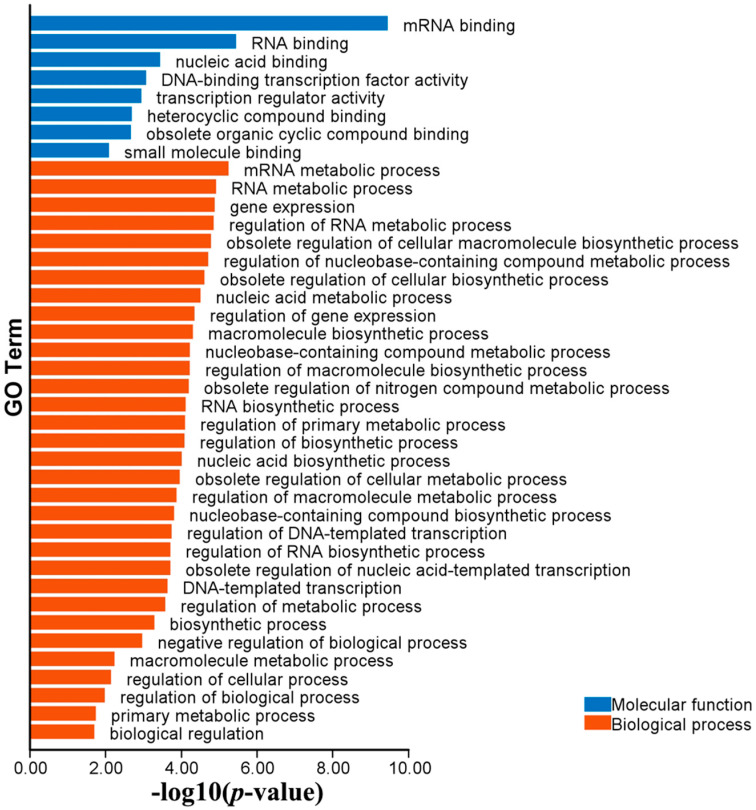
GO enrichment analyses of *MaC3Hs*. GO enrichment analysis of the mulberry CCCH genes. Based on the enrichment results, blue and red colors indicate the different categories.

To further elucidate the potential functions of the CCCH gene family in mulberry growth and development, Gene Ontology (GO) enrichment analysis was conducted to predict their participation in diverse biological processes. The identified GO terms included 32 biological processes and eight molecular functions (Figure 7, Table S8). Within the biological process category, the majority of genes were predicted to participate in metabolic processes, particularly RNA metabolism, nucleic acid metabolism and heterocyclic compound metabolism. Notably, these processes were primarily associated with eight genes: XM_024165356.1-0, XM_010110830.1-0, XM_010104852.2-0, XM_024162439.1-0, XM_010090035.2-0, XD_010098254.2-0, XM_010110597.2-0 and XM_010103125.2-0. For molecular function analysis, eight genes were found to be associated with several key functions, including mRNA binding (GO:0003729), RNA binding (GO:0003723), nucleic acid binding (GO:0003676), heterocyclic compound binding (GO:1901363), obsolete organic cyclic compound binding (GO:0097159) and small molecule binding (GO:0036094).

### 2.7. MaC3H Gene Expression Following Drought Treatment

Previous research has demonstrated that CCCH genes are associated with plant responses to drought stress [11,32]. To determine whether *MaC3H* genes contribute to drought tolerance in mulberry, we conducted quantitative real-time PCR (qRT-PCR) to examine their expression patterns in roots, stems and leaves of seedlings subjected to drought for 0, 3, 6, 9 and 12 days. Specific primers used for amplification are provided in Table S9. Based on a phylogenetic analysis of previously reported drought-responsive CCCH genes and *MaC3H* genes, 17 representative genes were selected for qRT-PCR analysis (Figure S1).

**Figure 8:**
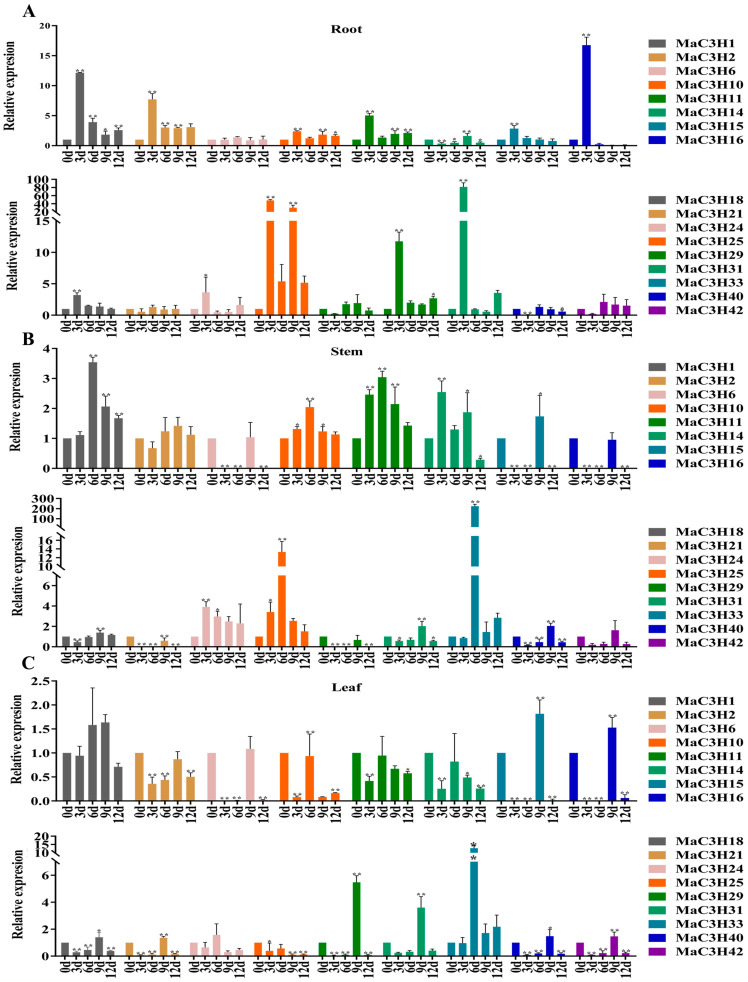
The expression of *MaC3H* gene in different parts under drought stress treatment. (**A**) Expression in the roots. (**B**) Expression in the stems. (**C**) Expression in leaves. *Y*-axis: relative expression levels; *X*-axis: the time course of drought stress treatments. Statistical significance was determined using a paired Student’s *t*-test. The mean ± standard error from the mean (SE) of at least three replicates is presented, and significant differences relative to controls are indicated at * *p  *<  0.05 and ** *p  *<  0.01.

Seventeen genes were upregulated in the roots at various time points following drought treatment, although *MaC3H6*, *MaC3H21*, *MaC3H19* and *MaC3H42* showed no significant differences compared with the control (0 day) (Figure 8A). After three days of drought treatment, eleven genes (*MaC3H1*, *MaC3H2*, *MaC3H10*, *MaC3H11*, *MaC3H15*, *MaC3H16*, *MaC3H18*, *MaC3H24*, *MaC3H25*, *MaC3H31* and *MaC3H33*) reached peak expression levels. Among them, five genes exhibited more than a tenfold increase, and *MaC3H33* was upregulated over 70-fold relative to the control, showing a highly significant difference. Four genes (*MaC3H6*, *MaC3H21*, *MaC3H40* and *MaC3H42*) reached maximum expression on day 6, while two genes (*MaC3H14* and *MaC3H29*) peaked on day 9.

In the stems, the expression of three genes (*MaC3H16*, *MaC3H21* and *MaC3H29*) was suppressed following drought treatment (Figure 8B). On day 9, ten genes (*MaC3H2*, *MaC3H6*, *MaC3H15*, *MaC3H16*, *MaC3H18*, *MaC3H21*, *MaC3H29*, *MaC3H31*, *MaC3H40* and *MaC3H42*) reached their highest expression levels, whereas at other time points their expression was either lower than or comparable to that of the control. Additionally, five genes (*MaC3H1*, *MaC3H10*, *MaC3H11*, *MaC3H25* and *MaC3H33*) peaked on day 6 and then declined. Among them, three genes exhibited expression levels more than tenfold higher than the control, with *MaC3H33* showing an exceptionally high induction, exceeding 200-fold on day 6. Moreover, *MaC3H14* and *MaC3H24* peaked on day 3 and displayed significant differences compared with the control.

In the leaves, five genes (*MaC3H1*, *MaC3H10*, *MaC3H11* and *MaC3H33*) showed suppressed expression after drought treatment (Figure 8C). Conversely, eleven genes (*MaC3H1*, *MaC3H2*, *MaC3H6*, *MaC3H15*, *MaC3H16*, *MaC3H18*, *MaC3H21*, *MaC3H29*, *MaC3H31*, *MaC3H40* and *MaC3H42*) reached peak expression levels on day 9, but only *MaC3H29* and *MaC3H31* exhibited more than a twofold increase compared with the control. Additionally, six genes (*MaC3H10*, *MaC3H11*, *MaC3H14*, *MaC3H24*, *MaC3H25* and *MaC3H33*) reached maximum expression levels on day 6, with *MaC3H33* showing a ten-fold increase relative to the control, which was statistically significant. By analyzing the expression level of *MaC3Hs*, it was found that *MaC3H33* exhibited significant responses in different parts under drought stress. Therefore, we will further investigate this gene.

### 2.8. Subcellular Localization and Transcription Activation Assay

**Figure 9:**
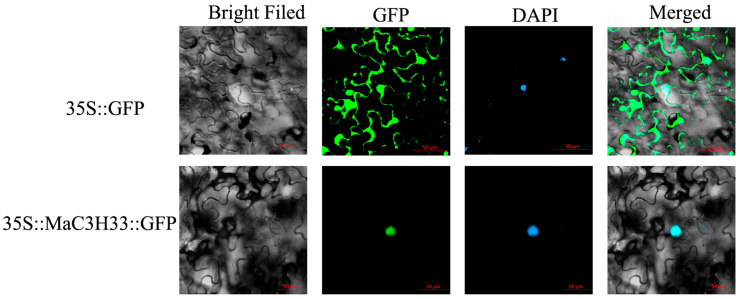
Subcellular localization analysis of *MaC3H33*. The images depicted the expression of MaC3H33-GFP fusion protein and GFP (control) in tobacco leaves. The green fluorescent signal of the GFP channel indicated the location of the expressed protein, the bright field showed the cell, the blue signal of DAPI showed the location of the expressed protein in the nucleus and Merged is a merged plot of the former. Scale bar = 50 µm.

*MaC3H33* was selected for subcellular localization and transcriptional activation analyses. To determine its subcellular localization, a MaC3H33-GFP fusion construct was generated and transiently expressed in Nicotiana benthamiana leaves. Fluorescence microscopy revealed that MaC3H33 was exclusively localized in the nucleus (Figure 9). 

**Figure 10:**
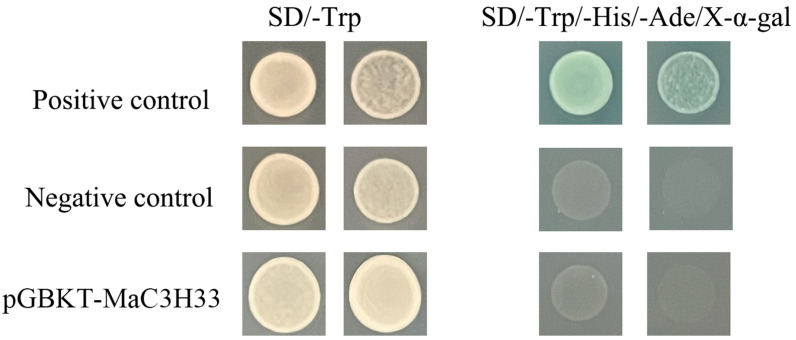
Transcriptional activation activity of MaC3H33. The fusion proteins of the GAL4 DNA binding (BD) and MaC3H33 were expressed in yeast strain.

For transcriptional activation analysis, the recombinant yeast expression vector MaC3H33-pGBKT7 was constructed and introduced into the yeast strain Y2H. As illustrated in Figure 10, MaC3H33-pGBKT7, together with both positive and negative controls, was able to grow on SD/−Trp medium. In contrast, on selective SD/−Trp/−His/−Ade/X-α-gal plates, only the positive control displayed growth and produced a blue coloration, whereas neither the negative control nor MaC3H33-pGBKT7 exhibited growth. These results demonstrate that MaC3H33 does not exhibit intrinsic transcriptional self-activation activity.

## 3. Discussion

CCCH-type zinc finger proteins have been characterized in several plant species, including *Arabidopsis* [28], rice [33], maize [34], cucumber (*Cucumis sativus* L.) [35], potato (*Solanum tuberosum* L.) [36] and others. These proteins have been implicated in diverse biological processes, such as plant growth, development and responses to both biotic and abiotic stresses. For example, a new non-tandem CCCH-type zinc finger gene (*PbdsZF*) was identified in Chinese white pear (*Pyrus bretschneideri*), which regulates lignin and stone cell formation [37]. However, information regarding the functions and regulatory roles of CCCH proteins in mulberry remains limited. To address this gap, we performed a genome-wide identification and characterization of CCCH proteins in mulberry, integrating bioinformatic analyses with qRT-PCR assays to explore their potential roles in developmental regulation and stress responses.

Our genome-wide survey uncovered 56 CCCH genes in mulberry. The number is comparable to that reported for *Arabidopsis* (68) and rice (73), but markedly lower than in maize (111), likely reflecting species-specific histories of whole-genome and lineage-specific duplication. The wide span of coding sequence (456–6318 bp) and predicted protein size (151–2105 aa) illustrates the structural plasticity typical of plant CCCH families and suggests functional diversification. Approximately 70% of MaC3H proteins were predicted to be exclusively nuclear, consistent with the view that many CCCH proteins act as nuclear RNA-binding regulators or transcriptional co-factors [38]. In *Arabidopsis*, *C3H15* regulates plant heat tolerance through transcriptional and post-transcriptional regulation [39]. A minority of *MaC3Hs* were predicted for dual or other organelle localization (chloroplast, endoplasmic reticulum and cytoplasm), suggesting possible organelle-specific functions in processes such as DNA and RNA synthesis. There are research reports that *ZC3H4* is an inhibitor of non-coding RNA (ncRNA) production, located at the critical intersection of DNA and RNA synthesis [40]. Chromosomal mapping revealed uneven distribution across all 14 chromosomes, with chromosome 6 harboring the highest number of genes. Tandem duplication events observed on chromosomes 1, 6 and 13, as well as four segmental duplication events, suggest that gene duplication has contributed to the expansion of the *MaC3H* gene family. The low Ka/Ks ratios (<1) of duplicated gene pairs suggest purifying selection has played a key role in maintaining functional integrity during evolution.

The structural organization of *MaC3H* genes, combined with their motif composition, reflects a high degree of evolutionary conservation while also indicating functional divergence within the mulberry genome. Previous studies have shown that the CCCH protein family exhibits high conservation in various plants such as rice [33] and carrots (*Daucus carota*) [41]. Conserved motif arrangements within the 13 subfamilies suggest shared regulatory or biochemical roles shaped by gene duplication. Subfamily-specific motifs, such as motif 9 in most A family members (except *MaC3H36*) and motif 6 in selected B family genes, indicate functional divergence, while the M family’s uniform motif patterns reflect strong selective constraints. Intron–exon analysis revealed most genes possessed 1–13 introns, though five A family members were intronless, suggesting potential for rapid transcriptional responses in stress or development. The 56 CCCH genes are divided into 13 subfamilies based on the phylogenetic tree, with uneven distribution of each subfamily (e.g., 10 genes in branch E and 2 genes in branches G, J, L and M), indicating that the regulation requirements of mulberry in response to developmental or stress adaptation processes lead to lineage specific expansion of certain branches [42]. In addition, *MaC3H5*, *MaC3H38* and *MaC3H56* are not classified as subfamilies. According to previous reports, there are also cases of uneven distribution of subfamilies and genes not belonging to families in plants such as *Arabidopsis* and rice [28]. *M. alba* has 45 orthologous pairs with *A. thaliana*, while only 13 pairs with *O. sativa* and 5 pairs with *Z. mays*. This supports the notion that dicot species like *A. thaliana* share a more recent common ancestor with *M. alba* than monocots such as *O. sativa* and *Z. mays*.

Among the cis-elements identified, phytohormone-responsive motifs such as ABRE (abscisic acid responsiveness), TCA-element (salicylic acid responsiveness) and TGA-element (auxin responsiveness) were the most prevalent, appearing in nearly all promoters except for a few (*MaC3H35*, *MaC3H46* and *MaC3H53*). This pattern implies that most *MaC3H* genes are likely to participate in hormone-regulated gene expression networks. Since hormones like ABA, auxin and SA are key regulators of plant development, growth, and defense, MaC3H proteins may participate in a broad spectrum of physiological activities, ranging from cell elongation and organ formation to senescence and immune responses. Their presence across diverse hormone pathways also suggests potential cross-talk among hormone signaling pathways, where *MaC3Hs* help coordinate and fine-tune overlapping hormonal signals under changing environmental or developmental contexts. Previous studies have shown that *AtC3H14* and *AtC3H15* can participate in plant biological processes by integrating hormones [3]. Although stress-responsive elements such as MBS (drought inducibility), ARE (anaerobic induction) and LTR (low-temperature responsiveness) were less abundant, their presence in several gene promoters suggests that some *MaC3Hs* might be inducible under specific abiotic stress conditions. CCCH proteins are commonly regulated by stress-responsive elements such as MBS, ARE, and LTR. In alfalfa, *MsC3H29* was significantly induced by drought in underground tissues [22]. In catalpa bungei, *CbuC3H24* and *CbuC3H58* showed the most significant response after cold stress response [43]. This selective distribution points to a functional divergence within the gene family. Certain members may have evolved to respond specifically to environmental stress cues, while others maintain roles more tightly linked to hormone signaling or development. This functional specialization aligns with common evolutionary patterns observed in multigene families. Further supporting these interpretations, the GO annotation analysis revealed that *MaC3H* genes are predominantly associated with RNA metabolic processes, particularly nucleic acid binding, RNA binding and mRNA binding. These molecular functions are characteristic of CCCH zinc finger proteins, which are known to mediate post-transcriptional regulation through mRNA decay, processing, export and translation control. This suggests that *MaC3Hs* may serve as downstream effectors of the signaling pathways indicated by the cis-element analysis, acting as downstream effectors that adjust post-transcriptional gene regulation in response to internal cues and environmental challenges. The convergence of transcriptional regulation, via hormone- and stress-responsive promoter elements, and post-transcriptional regulation, via RNA-related molecular functions, indicates that *MaC3Hs* have a multi-layered regulatory role. These genes likely act as both signal responders and modulators, helping to adjust gene expression rapidly and precisely during developmental transitions or under environmental stresses.

CCCH proteins are broadly implicated in stress responses, and several studies have reported their involvement in drought tolerance in other plant species. Fifty-seven CCCH genes were identified in the peppers (*Capsicum annuum* L.), and 6 genes were upregulated after drought treatment [44]. After drought treatment, the expression levels of 73 CCCH genes identified in rice, including *OsC3H5*, *OsC3H10* and *OsC3H38*, increased to varying degrees in different parts [33]. The CCCH zinc finger protein GmZF351 is an oil level regulator and can improve soybean drought resistance [32]. Expression profiling under drought stress provided critical insights into the functional relevance of *MaC3Hs*. qRT-PCR analysis of 17 selected genes across roots, stems and leaves showed diverse and dynamic expression patterns. In roots, many *MaC3H* genes were rapidly upregulated, particularly *MaC3H33*, which exhibited a dramatic >70-fold increase on day 3. Analysis of stems and leaves revealed that certain genes, including *MaC3H33* and *MaC3H9*, reached their highest expression levels on days 6 and 9, respectively. The findings indicate that *MaC3H* genes play critical roles in regulating mulberry’s dynamic response to drought stress, with certain members likely participating in the early stages of stress signaling, while others contribute to prolonged adaptation mechanisms. These results emphasize that *MaC3H* genes exhibit diverse expression across tissues, reflecting their multifunctionality and differential regulation. For instance, *MaC3H33* was highly upregulated in roots and stems but only moderately expressed in leaves, indicating a predominant role in subterranean tissues where drought stress is initially perceived. Conversely, the downregulation of *MaC3H10* and *MaC3H11* in leaves, despite their upregulation in roots, may suggest a shift in functional priorities, such as the redirection of energy and signaling resources away from aerial tissues. Similar tissue-specific regulation has been observed in pitaya (*Hylocereus polyrhizus*), where *HuTZF3* is highly expressed in roots and stems and enhances salt and heat tolerance [45]. Collectively, the data imply functional divergence within the *MaC3H* family, where specific genes (e.g., *MaC3H33*) respond rapidly to drought signals, while others (e.g., *MaC3H29*) play more prominent roles in maintaining long-term tolerance. The differential timing of peak expression across tissues further suggests that these genes are finely regulated, possibly through complex transcriptional and post-transcriptional mechanisms. The prominent induction of several genes in roots also reinforces the root’s central role in drought detection and systemic signaling.

## 4. Materials and Methods

### 4.1. Identification of CCCH Proteins in Mulberry Genome

The mulberry genome database was provided by Jiao et al. in the China National GeneBank DataBase (PRJNA597170 and PRJNA597172), and was used to identify members of CCCH protein family in mulberry [46]. Initially, CCCH proteins (PF00642) from the model plant *Arabidopsis thaliana* were used as queries to search for homologous sequences in the mulberry genome through BLASTP, applying an E-value threshold of 1e-005. Candidate sequences were subsequently verified by manual inspection using Pfam (https://pfam.sanger.ac.uk/, accessed on 10 July 2024) and SMART (http://smart.embl.de/, accessed on 10 July 2024). ExPASY provides information about molecular weight and isoelectric point [47]. And use TBtools 2.357 to obtain information on the average values of chromosome locus, CDS Length (bp), number of amino acids and molecular weight from the mulberry genome database [46,48]. Conserved motifs, including C-X8-C-X5-C-X3-H and C-X7-C-X5-C-X3-H, were identified in MaC3H protein sequences, and their sequence patterns were visualized as logos using the WebLogo platform [49].

### 4.2. Chromosomal Distribution Analysis

Physical locations of the *MaC3H* genes were determined using information provided in the mulberry genome annotation files. Chromosomal mapping was visualized using TBtools 2.357 software to assess their distribution across 14 chromosomes [48].

### 4.3. Phylogenetic Analysis and Gene Structure

The complete amino acid sequences of the MaC3H proteins were first aligned with ClustalX (version 1.83) [50]. To explore their evolutionary relationships and classify them into subfamilies, a phylogenetic tree was generated in MEGA using the neighbor-joining (NJ) method with 1000 bootstrap replicates. Gene Structure Display Server (GSDS) program was used to study the intron/exon structure of *MaC3H* genes and visualize it using TBtools 2.357 [51].

To identify conserved motifs, 56 amino acid sequences of MaC3H proteins were examined using the MEME tool (http://meme.sdsc.edu/meme/intro.html, accessed on 16 July 2024). The search was conducted with parameters specifying up to 10 motifs and an optimal motif width ranging from 25 to 100 residues [52].

### 4.4. Gene Duplication and Synteny Analysis

Collinearity analysis between *M. alba* and three representative species (*A. thaliana*, *O. sativa*, and *Z. mays*) was conducted using TBtools to identify both paralogous and orthologous gene pairs, thereby providing insights into their evolutionary relationships [48]. In addition, the nonsynonymous (Ka) and synonymous (Ks) substitution rates of duplicated gene pairs were calculated to evaluate the selective pressures acting on these genes [53].

### 4.5. Cis-Element Analysis

To investigate the regulatory features of *MaC3H* gene promoters, the 2000-bp sequences upstream of the transcription start site were analyzed using the PlantCARE database [54].

### 4.6. Gene Ontology (GO) Annotation

GO annotation of *MaC3H* genes was performed using the eggNOG-mapper and InterProScan to assign genes to biological process and molecular function categories. Detailed GO terms and associated genes are presented in Table S8 [55].

### 4.7. Plant Materials and Drought Treatment

Mulberry seedlings were grown under controlled conditions. To impose drought stress, irrigation was withheld, and samples from roots, stems and leaves were collected at 0, 3, 6, 9 and 12 days following treatment. For each sampling point, three independent biological replicates were obtained [56].

### 4.8. RNA Extraction and qRT-PCR

Total RNA was extracted from different plant tissues using the TaKaRa MiniBEST Plant RNA Extraction Kit according to the manufacturer’s instructions. First-strand cDNA was synthesized with the PrimeScript™ RT reagent kit (TaKaRa, Kyoto, Japan). Gene-specific primers were designed for each target sequence using Primer Premier 5.0 software (Table S9), with *MaActin* selected as the reference gene [57]. qRT-PCR was performed under the following cycling conditions: initial denaturation at 95 °C for 30 s, followed by 40 cycles of 95 °C for 10 s, 55 °C for 15 s, and 72 °C for 10 s. Relative transcript abundance was determined using the 2^−ΔΔCT^ method, with expression levels on day 0 normalized to a value of 1. Statistical significance was evaluated with SPSS 17.0, and data visualization was carried out using GraphPad Prism 5.

### 4.9. Subcellular Localization and Transactivation Activity

The coding sequence of *MaC3H33* was cloned into the pCAMBIA1305 vector to fuse with GFP under the control of the CaMV35S promoter. The recombinant construct was transferred into Agrobacterium tumefaciens strain GV3101 and subsequently infiltrated into Nicotiana benthamiana leaves. GFP signals were then detected using a confocal laser scanning microscope (LSM 800, ZEISS, Oberkochen, Germany).

The complete coding sequence (CDS) of MaC3H33 was inserted into the pGBKT7 vector and subsequently introduced into the Y_2_H-Gold yeast strain. The Y_2_H-Gold yeast strain was provided by Shanghai Weidi Biotechnology Co., Ltd (Shanghai, China) (https://www.weidibio.com/display.php?id=339, accessed on 24 July 2024). Transformants were first screened on SD medium lacking tryptophan (SD/−Trp) to confirm successful plasmid uptake. To evaluate whether MaC3H33 possessed transcriptional self-activation activity, the yeast colonies were further cultured on selective medium deficient in tryptophan, histidine and adenine, and supplemented with X-α-gal (SD/−Trp/−His/−Ade/X-α-gal). Colony growth and the appearance of blue coloration were then examined as indicators of transcriptional activation. In addition, positive (pGBKT7-53+pGADT7-T) and negative controls (pGBKT7) were set up.

## 5. Conclusions

In this study, we performed a genome-wide identification and characterization of 56 CCCH-type zinc finger genes (*MaC3Hs*) in mulberry. These genes were unevenly distributed across 14 chromosomes, with evidence of both tandem and segmental duplications driving family expansion. Phylogenetic and motif analyses classified them into 13 subfamilies, revealing conserved structural features alongside subfamily-specific variations. Promoter analysis indicated that most *MaC3Hs* contain hormone- and stress-responsive cis-acting elements, while GO enrichment linked them to RNA metabolism and nucleic acid binding, suggesting key roles in transcriptional and post-transcriptional regulation. Expression profiling under drought stress demonstrated that multiple *MaC3Hs* respond dynamically in roots, stems and leaves, with *MaC3H33* showing particularly strong induction. Subcellular localization confirmed that *MaC3H33* is nuclear, while transcriptional activation assays indicated it lacks self-activation ability, implying it may act with partner proteins. Together, these findings highlight the importance of *MaC3Hs* in hormone signaling, RNA metabolism and drought response. This work provides a foundation for functional studies of individual genes, with future efforts needed to validate their regulatory mechanisms and explore their potential in improving mulberry stress resilience. Importantly, the insights gained here may support molecular breeding strategies to enhance mulberry adaptation to environmental challenges.

## Data Availability

Regarding data availability, the following information is provided: The mulberry genome can be found on the China National GeneBank DataBase (https://db.cngb.org/, Number: PRJNA597170 and PRJNA597172, accessed on 27 August 2025). The following information can be found at https://phytozome-next.jgi.doe.gov/, 27 August 2025: *Arabidopsis*, rice, and maize.

## Supplementary Materials

The following supporting information can be downloaded at https://www.mdpi.com/article/10.3390/ijms26199490/s1.

## References

[1] Stege J.T., Guan X., Ho T., Beachy R.N., Barbas C.F. (2002). Controlling gene expression in plants using synthetic zinc finger transcription factors. Plant J..

[2] Blackshear P. (2002). Tristetraprolin and other CCCH tandem zinc-finger proteins in the regulation of mRNA turnover. Biochem. Soc. Trans..

[3] Wang Q., Song S., Lu X., Wang Y., Chen Y., Wu X., Tan L., Chai G. (2022). Hormone regulation of CCCH zinc finger proteins in plants. Int. J. Mol. Sci..

[4] Takatsuji H. (1998). Zinc-finger transcription factors in plants. Cell. Mol. Life Sci. CMLS.

[5] Kim D.H., Yamaguchi S., Lim S., Oh E., Park J., Hanada A., Kamiya Y., Choi G. (2008). SOMNUS, a CCCH-type zinc finger protein in *Arabidopsis*, negatively regulates light-dependent seed germination downstream of PIL5. Plant Cell.

[6] Seok H.-Y., Kim T., Lee S.-Y., Moon Y.-H. (2022). Non-TZF transcriptional activator AtC3H12 negatively affects seed germination and seedling development in *Arabidopsis*. Int. J. Mol. Sci..

[7] Yan Z., Jia J., Yan X., Shi H., Han Y. (2017). *Arabidopsis* KHZ1 and KHZ2, two novel non-tandem CCCH zinc-finger and K-homolog domain proteins, have redundant roles in the regulation of flowering and senescence. Plant Mol. Biol..

[8] Zhang D., Xu Z., Cao S., Chen K., Li S., Liu X., Gao C., Zhang B., Zhou Y. (2018). An uncanonical CCCH-tandem zinc-finger protein represses secondary wall synthesis and controls mechanical strength in rice. Mol. Plant.

[9] Seok H.-Y., Bae H., Kim T., Mehdi S.M.M., Nguyen L.V., Lee S.-Y., Moon Y.-H. (2021). Non-TZF protein ATC3H59/ZFWD3 is involved in seed germination, seedling development, and seed development, interacting with PPPDE family protein Desi1 in *Arabidopsis*. Int. J. Mol. Sci..

[10] Wang L., Wang R., Cai X., Zheng H., Huang Y., Li Y., Cui M., Lin M., Tang H. (2024). A loss-of-function mutation in OsTZF5 confers sensitivity to low temperature and effects the growth and development in rice. Plant Mol. Biol..

[11] Liu H., Xiao S., Sui S., Huang R., Wang X., Wu H., Liu X. (2022). A tandem CCCH type zinc finger protein gene CpC3H3 from *Chimonanthus praecox* promotes flowering and enhances drought tolerance in *Arabidopsis*. BMC Plant Biol..

[12] Chen F., Liu H.-L., Wang K., Gao Y.-M., Wu M., Xiang Y. (2020). Identification of CCCH zinc finger proteins family in moso bamboo (*Phyllostachys edulis*), and PeC3H74 confers drought tolerance to transgenic plants. Front. Plant Sci..

[13] Lan Y., Chen F., Zhang K., Wang L., Zhang S., Wu M., Xiang Y. (2023). The CCCH zinc finger protein PeC3H74 of Moso bamboo (*Phyllostachys edulis*) positively regulates drought and salinity tolerances in transgenic plants. Ind. Crops Prod..

[14] Zhang Q., Zhang J., Wei F., Fu X., Wei H., Lu J., Ma L., Wang H. (2023). The CCCH-type zinc-finger protein GhC3H20 enhances salt stress tolerance in *Arabidopsis* thaliana and cotton through ABA signal transduction pathway. Int. J. Mol. Sci..

[15] Bai H., Lin P., Li X., Liao X., Wan L., Yang X., Luo Y., Zhang L., Zhang F., Liu S. (2021). DgC3H1, a CCCH zinc finger protein gene, confers cold tolerance in transgenic chrysanthemum. Sci. Hortic..

[16] Cai J., Wang X., Wang Z., Sheng S., Tang F., Zhang Z. (2025). ZC3H13-mediated m6A modification ameliorates acute myocardial infarction through preventing inflammation, oxidative stress and ferroptosis by targeting lncRNA93358: ZC3H13 ameliorates AMI by inhibiting lncRNA93358 and ferroptosis. Inflammation.

[17] Selvaraj M.G., Jan A., Ishizaki T., Valencia M., Dedicova B., Maruyama K., Ogata T., Todaka D., Yamaguchi-Shinozaki K., Nakashima K. (2020). Expression of the CCCH-tandem zinc finger protein gene OsTZF5 under a stress-inducible promoter mitigates the effect of drought stress on rice grain yield under field conditions. Plant Biotechnol. J..

[18] Seok H.-Y., Lee S.-Y., Nguyen L.V., Bayzid M., Jang Y., Moon Y.-H. (2024). AtC3H3, an *Arabidopsis* Non-TZF gene, enhances salt tolerance by increasing the expression of both ABA-dependent and-independent stress-responsive genes. Int. J. Mol. Sci..

[19] Zhu J.-K. (2002). Salt and drought stress signal transduction in plants. Annu. Rev. Plant Biol..

[20] Zhu J.-K. (2016). Abiotic stress signaling and responses in plants. Cell.

[21] Grondin A., Natividad M.A., Ogata T., Jan A., Gaudin A.C., Trijatmiko K.R., Liwanag E., Maruyama K., Fujita Y., Yamaguchi-Shinozaki K. (2024). A case study from the overexpression of OsTZF5, encoding a CCCH tandem zinc finger protein, in rice plants across nineteen yield trials. Rice.

[22] Dong X., Han B., Chen J., Luo D., Zhou Q., Liu Z. (2024). Multiomics analyses reveal MsC3H29 positively regulates flavonoid biosynthesis to improve drought resistance of autotetraploid cultivated alfalfa (*Medicago sativa* L.). J. Agric. Food Chem..

[23] Li D., Yang J., Pak S., Zeng M., Sun J., Yu S., He Y., Li C. (2022). PuC3H35 confers drought tolerance by enhancing lignin and proanthocyanidin biosynthesis in the roots of *Populus ussuriensis*. New Phytol..

[24] Guo C., Chen L., Cui Y., Tang M., Guo Y., Yi Y., Li Y., Liu L., Chen L. (2022). RNA binding protein OsTZF7 traffics between the nucleus and processing bodies/stress granules and positively regulates drought stress in rice. Front. Plant Sci..

[25] Zhang Z.-A., Liu J.-Y., Tian J.-L., Khurshid M., Yan C.-H., Herman R.A., Gong L.-C., Wang J. (2025). Chemical analysis of mulberry (*Morus alba* L.) leaves treated with jasmonates on nutrition composition and biological activity. Food Chem..

[26] Kewcharoenwong P., Sompornpailin K. (2025). Novel hybrid spun silk yarn developed from Eri and mulberry silks under industrial level and its characterizations. J. Nat. Fibers.

[27] Ren Y., Guo G., Wang Z., Zhu L., Geng B. (2025). Response of yield and protein content of forage mulberry to irrigation in north china plain. Agronomy.

[28] Wang D., Guo Y., Wu C., Yang G., Li Y., Zheng C. (2008). Genome-wide analysis of CCCH zinc finger family in *Arabidopsis* and rice. BMC Genom..

[29] Chai G., Hu R., Zhang D., Qi G., Zuo R., Cao Y., Chen P., Kong Y., Zhou G. (2012). Comprehensive analysis of CCCH zinc finger family in poplar (*Populus trichocarpa*). BMC Genom..

[30] Xu G., Guo C., Shan H., Kong H. (2012). Divergence of duplicate genes in exon–intron structure. Proc. Natl. Acad. Sci. USA.

[31] Liu H., Gao Y., Wang L., Lan Y., Wu M., Yan H., Xiang Y. (2022). Identification and expression analysis of AP2/ERF superfamily in pecan (*Carya illinoensis*). Sci. Hortic..

[32] Wei W., Lu L., Bian X.H., Li Q.T., Han J.Q., Tao J.J., Yin C.C., Lai Y.C., Li W., Bi Y.D. (2023). Zinc-finger protein GmZF351 improves both salt and drought stress tolerance in soybean. J. Integr. Plant Biol..

[33] Wang Z., Li S., Wu H., Huang L., Fu L., Zhan C., Lu X., Yang L., Dai L., Zeng D. (2025). Identification and expression analysis of CCCH zinc finger family genes in *Oryza sativa*. Genes.

[34] Peng X., Zhao Y., Cao J., Zhang W., Jiang H., Li X., Ma Q., Zhu S., Cheng B. (2012). CCCH-type zinc finger family in maize: Genome-wide identification, classification and expression profiling under abscisic acid and drought treatments. PLoS ONE.

[35] Uddin S., Gull S., Hussain H.A., Mahmood U., Qasim M., Kamal F., Gaafar A.-R.Z., Aghayeva S., Iqbal R., Yang X. (2025). Genome-wide identification, characterization and expression analysis of CsC3H gene family in cucumber (*Cucumis sativus* L.) under various abiotic stresses. Plant Sci..

[36] Deng Z., Yang Z., Liu X., Dai X., Zhang J., Deng K. (2023). Genome-wide identification and expression analysis of C3H zinc finger family in potato (*Solanum tuberosum* L.). Int. J. Mol. Sci..

[37] Cao Y., Feng X., Ding B., Huo H., Abdullah M., Hong J., Jiang L., Wang H., Li R., Cai Y. (2025). Gap-free genome assemblies of two *Pyrus bretschneideri* cultivars and GWAS analyses identify a CCCH zinc finger protein as a key regulator of stone cell formation in pear fruit. Plant Commun..

[38] Zhang H., Dai Z., Zhang X., Shang M., Gao X., Ma R., Zhao L., Zhang X., Liu Q., Zhai H. (2025). Natural allelic variations in IbCHYR1–IbZnFR complex regulate fusarium root rot resistance in sweet potato. Adv. Sci..

[39] Chai G., Liu H., Zhang Y., Wang C., Xu H., He G., Meng J., Tang X., Wang D., Zhou G. (2024). Integration of C3H15-mediated transcriptional and post-transcriptional regulation confers plant thermotolerance in *Arabidopsis*. Plant J..

[40] Frey Y., Goehring L., Haj M., Rona G., Fijen C., Pagano M., Huang T.T., Rothenberg E., Ziv Y., Shiloh Y. (2025). ZC3H4 safeguards genome integrity by preventing transcription-replication conflicts at noncoding RNA loci. Sci. Adv..

[41] Yıldırım B.Ş., Öztürk Z.N. (2025). Genome-wide in silico identification, classification, and evolutionary analysis of putative abiotic stress-related CCCH genes in carrot. Plant Mol. Biol. Report..

[42] Zheng L., Dai H., Mu Y., Li J., Cheng Y., Han J. (2025). Genome-wide identification and expression analysis of C3H gene family in melon. Front. Plant Sci..

[43] Bao P., Sun J., Qu G., Yan M., Cheng S., Ma W., Wang J., Hu R. (2024). Identification and expression analysis of CCCH gene family and screening of key low temperature stress response gene CbuC3H24 and CbuC3H58 in Catalpa bungei. BMC Genom..

[44] Tang W., Hao Y., Ma X., Shi Y., Dang Y., Dong Z., Zhao Y., Zhao T., Zhu S., Zhang Z. (2023). Genome-wide analysis and identification of stress-responsive genes of the CCCH zinc finger family in *Capsicum annuum* L.. Front. Plant Sci..

[45] Xu W., Jian S., Li J., Wang Y., Zhang M., Xia K. (2023). Genomic identification of CCCH-Type zinc finger protein genes reveals the role of HuTZF3 in tolerance of heat and salt stress of pitaya (*Hylocereus polyrhizus*). Int. J. Mol. Sci..

[46] Jiao F., Luo R., Dai X., Liu H., Yu G., Han S., Lu X., Su C., Chen Q., Song Q. (2020). Chromosome-level reference genome and population genomic analysis provide insights into the evolution and improvement of domesticated mulberry (*Morus alba*). Mol. Plant.

[47] Gasteiger E., Gattiker A., Hoogland C., Ivanyi I., Appel R.D., Bairoch A. (2003). ExPASy: The proteomics server for in-depth protein knowledge and analysis. Nucleic Acids Res..

[48] Chen C., Wu Y., Li J., Wang X., Zeng Z., Xu J., Liu Y., Feng J., Chen H., He Y. (2023). TBtools-II: A “one for all, all for one” bioinformatics platform for biological big-data mining. Mol. Plant.

[49] Crooks G.E., Hon G., Chandonia J.-M., Brenner S.E. (2004). Weblogo: A sequence logo generator. Genome Res..

[50] Thompson J.D., Gibson T.J., Plewniak F., Jeanmougin F., Higgins D.G. (1997). The CLUSTAL_X windows interface: Flexible strategies for multiple sequence alignment aided by quality analysis tools. Nucleic Acids Res..

[51] Hu B., Jin J., Guo A.-Y., Zhang H., Luo J., Gao G. (2015). GSDS 2.0: An upgraded gene feature visualization server. Bioinformatics.

[52] Ma J., Wang Q., Sun R., Xie F., Jones D.C., Zhang B. (2014). Genome-wide identification and expression analysis of TCP transcription factors in Gossypium raimondii. Sci. Rep..

[53] Blanc G., Wolfe K.H. (2004). Widespread paleopolyploidy in model plant species inferred from age distributions of duplicate genes. Plant Cell.

[54] Liu Q., Wang H., Zhang Z., Wu J., Feng Y., Zhu Z. (2009). Divergence in function and expression of the NOD26-like intrinsic proteins in plants. BMC Genom..

[55] Cantalapiedra C.P., Hernández-Plaza A., Letunic I., Bork P., Huerta-Cepas J. (2021). eggNOG-mapper v2: Functional annotation, orthology assignments, and domain prediction at the metagenomic scale. Mol. Biol. Evol..

[56] Su X., Zhao M., Zhou R., Xu C., Zhang R., Li R., Wang T. (2025). The mulberry WRKY transcription factor MaWRKYIIc7 participates in regulating plant drought stress tolerance. Int. J. Mol. Sci..

[57] Zhou F., Xu L., Shi C., Wu F., Yang S. (2024). Identification of the optimal quantitative RT-PCR reference gene for paper mulberry (*Broussonetia papyrifera*). Curr. Issues Mol. Biol..

