# Supplementary figures and images for: Identification and Expression Analysis of CCCH Zinc Finger Proteins in Mulberry (Morus alba)
Source: Int J Mol Sci. 2025 Sep 28;26(19):9490. doi: 10.3390/ijms26199490 (PMC12524820; doi:10.3390/ijms26199490)

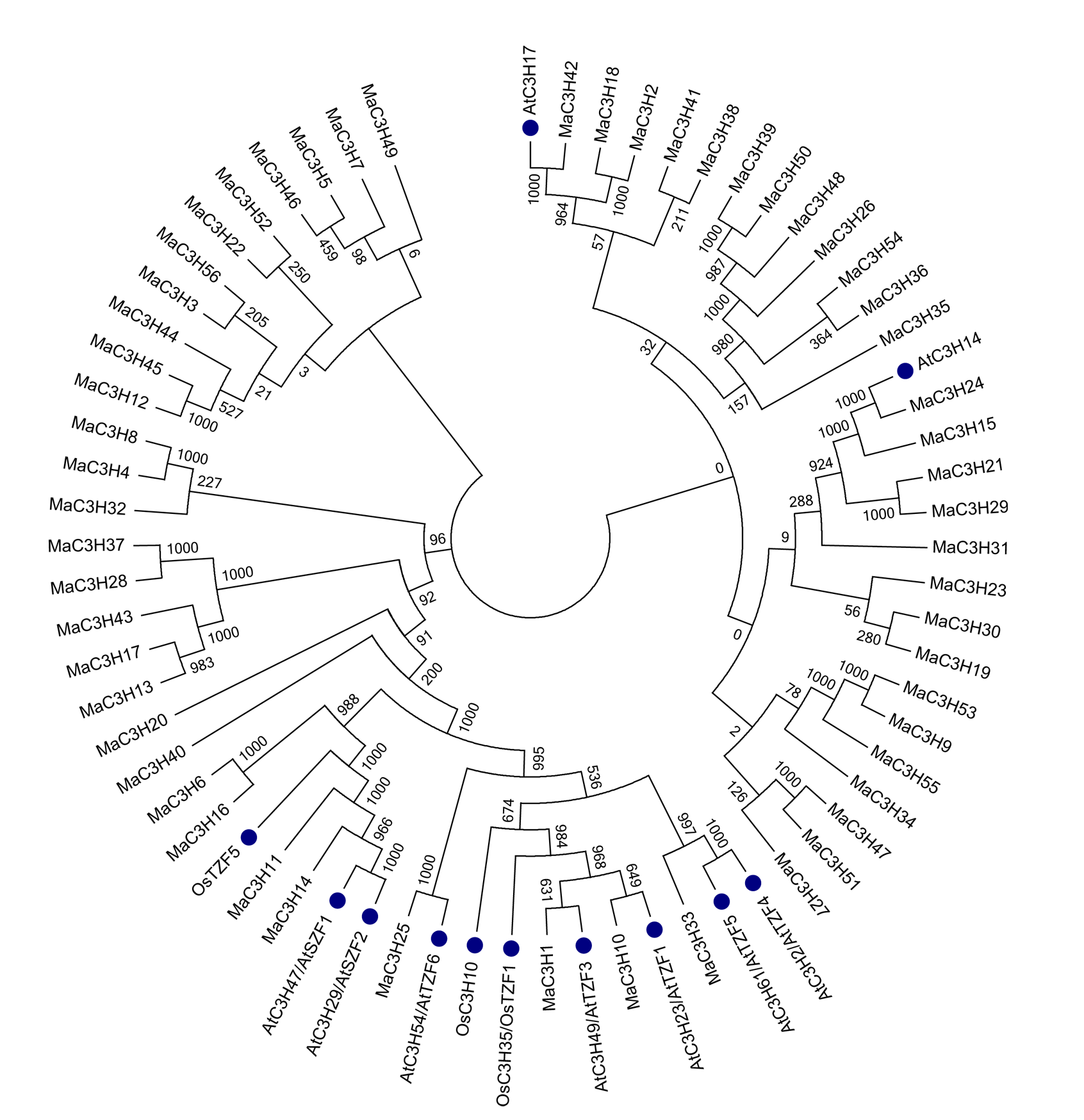

Supplement: Supplementary file 1 [file ijms-26-09490-s001.zip › Supplementary Materials/Figure S1.tif]
