# Supplementary material for: Identification and Expression Analysis of CCCH Zinc Finger Proteins in Mulberry (Morus alba)
Source: Int J Mol Sci. 2025 Sep 28;26(19):9490. doi: 10.3390/ijms26199490 (PMC12524820; doi:10.3390/ijms26199490)
Supplement: Supplementary file 1 [file ijms-26-09490-s001.zip › Supplementary Materials/Table S1.doc]

Table S1 Details of the identified MaC3H genes.

| **Name** | **Sequence ID** | **Chromosome** | **Locus** | **CDS Length (bp)** | **Number of Amino Acid** | **Molecular Weight** | **Theoretical pI** | **Subcelluar localization** |
| --- | --- | --- | --- | --- | --- | --- | --- | --- |
| MaC3H1 | XM_010088599.2-0 | Chr9 | 15698995-15700811 | 1164 | 387 | 42605 | 9.23 | Nucleus |
| MaC3H2 | XM_010089085.2-0 | Chr6 | 21011682-21015123 | 915 | 304 | 32010.11 | 9.31 | Chloroplast |
| MaC3H3 | XM_010090025.2-0 | Chr1 | 24146664-24150165 | 1629 | 542 | 61772.96 | 6.25 | Nucleus |
| MaC3H4 | XM_010090035.2-0 | Chr1 | 24059287-24061584 | 1047 | 348 | 38886.7 | 7.06 | Nucleus |
| MaC3H5 | XM_010094375.2-0 | Chr4 | 11954830-11963876 | 2145 | 714 | 77726.57 | 6.15 | Nucleus |
| MaC3H6 | XM_010094683.2-0 | Chr3 | 13075722-13080204 | 2208 | 735 | 80018.72 | 6.06 | Nucleus |
| MaC3H7 | XM_010097375.2-0 | Chr7 | 13873100-13877400 | 2073 | 690 | 76900.7 | 5.89 | Nucleus |
| MaC3H8 | XM_010098254.2-0 | Chr12 | 9215071-9218851 | 1086 | 361 | 39044.74 | 7.09 | Nucleus |
| MaC3H9 | XM_010098550.2-0 | Chr3 | 18506830-18510676 | 984 | 327 | 38150.06 | 9.63 | Chloroplast/Nucleus |
| MaC3H10 | XM_010098806.2-0 | Chr5 | 17835254-17836847 | 1041 | 346 | 38028.4 | 7.56 | Nucleus |
| MaC3H11 | XM_010099323.2-0 | Chr6 | 12932122-12935451 | 2124 | 707 | 76268.89 | 6.1 | Nucleus |
| MaC3H12 | XM_010100754.1-0 | Chr7 | 9826553-9830205 | 1251 | 416 | 46145.67 | 8.09 | Nucleus |
| MaC3H13 | XM_010102947.2-0 | Chr6 | 20052437-20056951 | 2064 | 687 | 75417.03 | 5.83 | Chloroplast/Nucleus |
| MaC3H14 | XM_010103125.2-0 | Chr11 | 6972586-6975463 | 2010 | 669 | 72716.92 | 7.11 | Nucleus |
| MaC3H15 | XM_010103410.2-0 | Chr14 | 9917848-9921747 | 1083 | 360 | 40948.47 | 9.68 | Nucleus |
| MaC3H16 | XM_010104042.1-0 | Chr6 | 20273639-20275528 | 1890 | 629 | 69136.13 | 6.91 | Endoplasmic reticulum/Nucleus |
| MaC3H17 | XM_010104074.2-0 | Chr6 | 20062476-20065911 | 1707 | 568 | 63826.44 | 5.49 | Chloroplast/Nucleus |
| MaC3H18 | XM_010104241.2-0 | Chr3 | 12292631-12296311 | 894 | 297 | 30953.41 | 9.52 | Nucleus |
| MaC3H19 | XM_010104852.2-0 | Chr1 | 56586114-56606587 | 975 | 324 | 36522.39 | 6.83 | Nucleus |
| MaC3H20 | XM_010106340.2-0 | Chr6 | 13218304-13221073 | 1275 | 424 | 50157.46 | 8.4 | Chloroplast/Nucleus |
| MaC3H21 | XM_010106959.2-0 | Chr5 | 12327611-12331598 | 1341 | 446 | 48671.5 | 6.12 | Nucleus |
| MaC3H22 | XM_010107070.2-0 | Chr8 | 11633571-11638190 | 2856 | 951 | 104042.04 | 8.33 | Nucleus |
| MaC3H23 | XM_010110511.2-0 | Chr10 | 1843779-1849048 | 2793 | 930 | 100842.62 | 6.73 | Chloroplast/Nucleus |
| MaC3H24 | XM_010110597.2-0 | Chr10 | 16443851-16445268 | 1020 | 339 | 37844.19 | 6.64 | Nucleus |
| MaC3H25 | XM_010110830.1-0 | Chr11 | 5402876-5403694 | 819 | 272 | 30891.58 | 8.37 | Nucleus |
| MaC3H26 | XM_010111918.2-0 | Chr10 | 15625862-15630317 | 1305 | 434 | 47086.62 | 8.53 | Nucleus |
| MaC3H27 | XM_010112589.2-0 | Chr14 | 12986700-12990986 | 1485 | 494 | 55156.86 | 8.14 | Chloroplast/Nucleus |
| MaC3H28 | XM_010114467.2-0 | Chr10 | 14778110-14782752 | 1680 | 559 | 63660.96 | 6.33 | Chloroplast |
| MaC3H29 | XM_010115069.2-0 | Chr13 | 1761703-1765449 | 1518 | 505 | 55442.48 | 8 | Nucleus |
| MaC3H30 | XM_024161399.1-0 | Chr9 | 15167242-15172068 | 1821 | 606 | 67467.15 | 7.15 | Chloroplast |
| MaC3H31 | XM_024161406.1-0 | Chr9 | 15027665-15034317 | 3045 | 1014 | 111079.92 | 5.87 | Nucleus |
| MaC3H32 | XM_024161979.1-0 | Chr13 | 14964666-14972998 | 2787 | 928 | 103156.54 | 8.92 | Nucleus |
| MaC3H33 | XM_024162439.1-0 | Chr10 | 9992927-9994374 | 1110 | 369 | 41187.58 | 8.16 | Nucleus |
| MaC3H34 | XM_024163299.1-0 | Chr5 | 19748563-19757919 | 6318 | 2105 | 232685.74 | 8.3 | Nucleus |
| MaC3H35 | XM_024163451.1-0 | Chr7 | 19100871-19106299 | 1119 | 372 | 40257.39 | 7.55 | Nucleus |
| MaC3H36 | XM_024165356.1-0 | Chr11 | 15129980-15153005 | 1506 | 501 | 53705.44 | 8 | Nucleus |
| MaC3H37 | XM_024166169.1-0 | Chr1 | 5647717-5652088 | 1674 | 557 | 62893.5 | 6.17 | Chloroplast |
| MaC3H38 | XM_024166795.1-0 | Chr6 | 16350633-16359820 | 2580 | 859 | 92733.09 | 5.41 | Cytoplasm |
| MaC3H39 | XM_024166836.1-0 | Chr2 | 4365438-4369716 | 1461 | 486 | 51365.41 | 8.62 | Nucleus |
| MaC3H40 | XM_024166952.1-0 | Chr6 | 10731631-10743737 | 456 | 151 | 17106.22 | 8.59 | Nucleus |
| MaC3H41 | XM_024168958.1-0 | Chr11 | 10386234-10391634 | 1092 | 363 | 41428.68 | 5.38 | Nucleus |
| MaC3H42 | XM_024170360.1-0 | Chr10 | 6752059-6755840 | 2166 | 721 | 79623.92 | 5.39 | Nucleus |
| MaC3H43 | XM_024170525.1-0 | Chr8 | 1111579-6755840 | 2214 | 737 | 80525.74 | 6.25 | Nucleus |
| MaC3H44 | XM_024170829.1-0 | Chr8 | 326562-329969 | 1248 | 415 | 46897.98 | 5.91 | Nucleus |
| MaC3H45 | XM_024172506.1-0 | Chr11 | 3823835-3828018 | 1299 | 432 | 46889.15 | 7.86 | Nucleus |
| MaC3H46 | XM_024174220.1-0 | Chr10 | 8594071-8610657 | 1308 | 435 | 48956.69 | 6.73 | Nucleus |
| MaC3H47 | XM_024174323.1-0 | Chr7 | 10990996-11009896 | 4605 | 1534 | 168531.71 | 4.93 | Nucleus |
| MaC3H48 | XM_024175206.1-0 | Chr6 | 19661652-19666533 | 1347 | 448 | 49135.42 | 8.48 | Nucleus |
| MaC3H49 | XM_024175284.1-0 | Chr4 | 5594492-5605826 | 2220 | 739 | 81223.6 | 4.98 | Nucleus |
| MaC3H50 | XM_024176256.1-0 | Chr7 | 8313889-8318316 | 1401 | 466 | 49897.22 | 9.02 | Nucleus |
| MaC3H51 | XM_024177121.1-0 | Chr13 | 1920457-1927586 | 4317 | 1438 | 157806.09 | 5.75 | Nucleus |
| MaC3H52 | augTM-XM_010109983.2-0 | Chr5 | 19284022-19287209 | 1512 | 503 | 56243.93 | 5.44 | Nucleus |
| MaC3H53 | augTM-XM_024164319.1-0 | Chr5 | 21372003-21376054 | 840 | 279 | 33286.99 | 9.34 | Chloroplast/Nucleus |
| MaC3H54 | augTM-XM_024169243.1-0 | Chr8 | 3508201-3512127 | 1722 | 573 | 62445.62 | 4.73 | Nucleus |
| MaC3H55 | augTM-XM_024170042.1-0 | Chr2 | 28445935-28459499 | 2580 | 859 | 100836.38 | 6.62 | Nucleus |
| MaC3H56 | augTM-XM_024172267.1-0 | Chr7 | 8834560-8841195 | 3063 | 1020 | 115653.86 | 6.3 | Nucleus |
