# Supplementary material for: Identification and Expression Analysis of CCCH Zinc Finger Proteins in Mulberry (Morus alba)
Source: Int J Mol Sci. 2025 Sep 28;26(19):9490. doi: 10.3390/ijms26199490 (PMC12524820; doi:10.3390/ijms26199490)
Supplement: Supplementary file 1 [file ijms-26-09490-s001.zip › Supplementary Materials/Table S2.doc]

Table S2 Detailed information on conserved amino acid sequences and motif lengths.

| **Motif** | **Length (aa)** | **Best possible match** | **Sequence logos of the MaC3Hs motifs** |
| --- | --- | --- | --- |
| 1 | 25 | FKAKCCKYFRKGSCKRGDSCRFAHG |  |
| 2 | 46 | IYSTDEFRMYEFKIRPCSRARSHDWTECPFAHPGEKARRRDPRKYH |  |
| 3 | 80 | SRQIYLTFPAESTFREEDVSNYFNKFGPVQDVRIPYQQKRMFGFVTFVYPETVKFILAKGNPHFVCDARVLVKPYKEKGK |  |
| 4 | 49 | VFECWLHPARYRTRLCKDGTSCRRRVCFFAHTPEZLRPLYASTGPAVPS |  |
| 5 | 25 | RVCPYYLKTGTCKFGDKCKFLHPLP |  |
| 6 | 63 | MDSYEATKIVFSRIQSIEPENASKIMGYLLIQDHGEKEMIRLAFGPEALJHSLILKAKTELGL |  |
| 7 | 30 | TPERPGQPECKYFMKTGDCPFGERCKFLHP |  |
| 8 | 35 | LNPYGLPLRPGZPECSYYSKYGICKFGPTCKFDHP |  |
| 9 | 77 | MGFEERTPLMVAAMYGSVDVLKYILELGKVDVNRSCGSDKSTALHCAAAGGSAAAIDVVKLLLAAGADPNSLDSNGN |  |
| 10 | 30 | LPERPGEPDCIFYLRTGYCKFGSTCKFNHP |  |
