# Supplementary material for: Identification and Expression Analysis of CCCH Zinc Finger Proteins in Mulberry (Morus alba)
Source: Int J Mol Sci. 2025 Sep 28;26(19):9490. doi: 10.3390/ijms26199490 (PMC12524820; doi:10.3390/ijms26199490)
Supplement: Supplementary file 1 [file ijms-26-09490-s001.zip › Supplementary Materials/Table S3.doc]

| Paralogous pairs | ka | ks | Ka/ks |
| --- | --- | --- | --- |
| XM_010110597.2-0/XM_010103410.2-0 | 0.5125229941801931 | 1.3742202394128458 | 0.37295549831166475 |
| XM_024166836.1-0/XM_024176256.1-0 | 0.28844943163794207 | 1.205680158460293 | 0.2392420822503248 |
| XM_010103125.2-0/XM_010099323.2-0 | 0.3071 | 1.8388 | 0.167011094 |
| XM_010098806.2-0/XM_010088599.2-0 | 0.4038 | 1.4669 | 0.275274388 |

Table S3 Ka, Ks and Ka/Ks values for the CCCH genes in *M. alba*.
