# Supplementary material for: Identification and Expression Analysis of CCCH Zinc Finger Proteins in Mulberry (Morus alba)
Source: Int J Mol Sci. 2025 Sep 28;26(19):9490. doi: 10.3390/ijms26199490 (PMC12524820; doi:10.3390/ijms26199490)
Supplement: Supplementary file 1 [file ijms-26-09490-s001.zip › Supplementary Materials/Table S4.doc]

Table S4 Ka, Ks and Ka/Ks values for the CCCH genes in *M. alba* and *A. thaliana*.

| Orthologous pairs | Ka | Ks | Ka/Ks |
| --- | --- | --- | --- |
| XM_010088599.2-0/AT4G29190 | 1.6727 | NaN | NaN |
| XM_010094375.2-0/AT1G30460 | 0.21002593107898235 | 2.047310478534619 | 0.10258626294401145 |
| XM_010097375.2-0/AT4G38890 | 0.23261619622787946 | 2.0396392989392216 | 0.11404771243075126 |
| XM_010098254.2-0/AT1G32360 | 0.2348130047203733 | NaN | NaN |
| XM_010098254.2-0/AT2G35430 | 0.3274456639322618 | NaN | NaN |
| XM_010098550.2-0/AT3G44785 | 0.21361089663925945 | NaN | NaN |
| XM_010098806.2-0/AT2G19810 | 0.33264313550751723 | NaN | NaN |
| XM_010098806.2-0/AT2G25900 | 0.3396135227652933 | 1.760615042567834 | 0.1928948205906337 |
| XM_010099323.2-0/AT5G58620 | 0.3005 | 3.1012 | 0.096897975 |
| XM_010102947.2-0/AT1G51520 | 0.47773268181966777 | 2.1919188332940704 | 0.21795181215798906 |
| XM_010102947.2-0/AT3G21100 | 0.3766364986365901 | 1.5612203855936604 | 0.24124492743756515 |
| XM_010102947.2-0/AT5G12440 | 0.40173891924876204 | NaN | NaN |
| XM_010103125.2-0/AT2G40140 | 0.2738475242537119 | 3.9074258179553216 | 0.07008387030544085 |
| XM_010103125.2-0/AT3G55980 | 0.27999500928374543 | 2.667462822894601 | 0.10496678974513635 |
| XM_010103410.2-0/AT1G66810 | 0.5209335203103003 | NaN | NaN |
| XM_010104042.1-0/AT5G12850 | 0.3738098807823261 | 2.108168666147525 | 0.1773149780588607 |
| XM_010106340.2-0/AT3G47120 | 0.35743261750398914 | 1.9782149179666622 | 0.18068442122121978 |
| XM_010110597.2-0/AT1G66810 | 0.4318438021579031 | 4.565507060531832 | 0.09458835490391246 |
| XM_010110830.1-0/AT5G07500 | 0.346222282314795 | 3.1724302675199425 | 0.10913471790365163 |
| XM_010111918.2-0/AT1G04990 | 0.3687599190170966 | 1.3929058279236328 | 0.2647414574801493 |
| XM_010115069.2-0/AT1G19860 | 0.4734613926770564 | NaN | NaN |
| XM_010115069.2-0/AT3G51180 | 0.578593538743152 | 4.171784738917105 | 0.1386920886271186 |
| XM_010115069.2-0/AT5G66270 | 0.6204326709660271 | 2.0252718033869668 | 0.3063453853099843 |
| XM_024161406.1-0/AT5G56930 | 0.5537654801674652 | 2.49688977519796 | 0.22178210895335226 |
| XM_024162439.1-0/AT1G03790 | 0.3509178156841217 | NaN | NaN |
| XM_024162439.1-0/AT5G44260 | 0.37955373832651035 | NaN | NaN |
| XM_024163299.1-0/AT1G21580 | 0.6191399674377347 | 1.922075427663446 | 0.3221205362322262 |
| XM_024166795.1-0/AT2G28450 | 0.30243432142805793 | 2.1522767467824666 | 0.1405183240864263 |
| XM_024166836.1-0/AT3G06410 | 0.27809931887879064 | 2.4958354604697632 | 0.11142534164750072 |
| XM_024166836.1-0/AT5G18550 | 0.3080395943727401 | 2.0074330722459823 | 0.1534494965892413 |
| XM_024170525.1-0/AT3G51950 | 0.2955328287585026 | 2.0105786846129563 | 0.14698893956263828 |
| XM_024170829.1-0/AT3G08505 | 0.2565069730226793 | NaN | NaN |
| XM_024172506.1-0/AT4G25440 | 0.20811906115952328 | 1.779901995155997 | 0.11692725876251574 |
| XM_024172506.1-0/AT5G51980 | 0.22050047686381352 | 1.9777608209890918 | 0.11148996103256799 |
| XM_024174220.1-0/AT3G21810 | 0.5741294059315449 | 1.7604774057629566 | 0.326121428228571 |
| XM_024175206.1-0/AT3G02830 | 0.1558436453355094 | 1.9849705413568488 | 0.07851181772651433 |
| XM_024175206.1-0/AT5G16540 | 0.23304947962531786 | 1.4560136253484126 | 0.16005995793449457 |
| XM_024175284.1-0/AT2G33835 | 0.652807531264739 | 1.7966575779626268 | 0.3633455474609744 |
| XM_024176256.1-0/AT2G47850 | 0.17189284428524473 | 1.2516542650067297 | 0.1373325279120273 |
| XM_024177121.1-0/AT2G18090 | 0.49343714824882257 | 2.3967482612674864 | 0.2058777537144745 |
| XM_024177121.1-0/AT3G51120 | 0.4194648756292543 | 2.283089103767707 | 0.1837268965705391 |
| augTM-XM_010109983.2-0/AT2G24830 | 0.2783497703354717 | 2.7206789651683807 | 0.10230893607774295 |
| augTM-XM_024169243.1-0/AT3G48440 | 0.35852548221772473 | 2.0414605168923345 | 0.1756220506108535 |
| augTM-XM_024169243.1-0/AT5G63260 | 0.4295224610079226 | 1.8510159309962255 | 0.23204687426798729 |
| augTM-XM_024172267.1-0/AT2G47680 | 0.2412393109899703 | 1.4781022766145884 | 0.16320880821758782 |
