# Supplementary material for: Identification and Expression Analysis of CCCH Zinc Finger Proteins in Mulberry (Morus alba)
Source: Int J Mol Sci. 2025 Sep 28;26(19):9490. doi: 10.3390/ijms26199490 (PMC12524820; doi:10.3390/ijms26199490)
Supplement: Supplementary file 1 [file ijms-26-09490-s001.zip › Supplementary Materials/Table S5.doc]

Table S5 Ka, Ks and Ka/Ks values for the CCCH genes in *M. alba* and *O. sativ*a.

| Orthologous pairs | Ka | Ks | Ka/Ks |
| --- | --- | --- | --- |
| XM_010088599.2-0/LOC_Os01g09620.1 | NaN | 1.6023 | NaN |
| XM_010088599.2-0/LOC_Os01g53650.1 | 2.7203 | 1.5908 | 1.710020116 |
| XM_010088599.2-0/LOC_Os05g10670.1 | 2.8865 | 1.7177 | 1.680444781 |
| XM_010088599.2-0/LOC_Os05g45020.1 | 2.4756 | 1.6583 | 1.492854128 |
| XM_010089085.2-0/LOC_Os02g10080.1 | 0.2084720338025954 | 2.5074775524324124 | 0.08314013961974029 |
| XM_024166169.1-0/LOC_Os07g39440.1 | 0.5248837211529775 | NaN | NaN |
| XM_024168958.1-0/LOC_Os01g61830.1 | 0.14055811857488765 | NaN | NaN |
| XM_024172506.1-0/LOC_Os02g45480.1 | 0.3017923197809375 | NaN | NaN |
| XM_024174220.1-0/LOC_Os06g46890.1 | 0.6665208746798712 | 1.874366332360699 | 0.35559797632537044 |
| XM_024177121.1-0/LOC_Os09g36090.1 | 0.5259627985754567 | NaN | NaN |
| augTM-XM_024164319.1-0/LOC_Os09g31482.1 | 0.18641801066086938 | 1.0520880125374312 | 0.17718860821469248 |
| augTM-XM_024169243.1-0/LOC_Os12g18120.1 | 0.5424946970436451 | 1.9374725290089618 | 0.28000123300903623 |
| augTM-XM_024172267.1-0/LOC_Os01g15300.1 | 0.3375356004120462 | 2.0715780097223058 | 0.16293646622426386 |
