# Supplementary material for: Identification and Expression Analysis of CCCH Zinc Finger Proteins in Mulberry (Morus alba)
Source: Int J Mol Sci. 2025 Sep 28;26(19):9490. doi: 10.3390/ijms26199490 (PMC12524820; doi:10.3390/ijms26199490)
Supplement: Supplementary file 1 [file ijms-26-09490-s001.zip › Supplementary Materials/Table S6.doc]

Table S6 Ka, Ks and Ka/Ks values for the CCCH genes in *M. alba* and *Z. mays*.

| Orthologous pairs | Ka | Ks | Ka/Ks |
| --- | --- | --- | --- |
| XM_010088599.2-0/GRMZM2G173124_T01 | 2.6829 | 1.403 | 1.912259444 |
| XM_010088599.2-0/GRMZM2G004795_T01 | 2.1463 | 1.2663 | 1.694938008 |
| XM_010089085.2-0/GRMZM2G056920_T01 | 0.30585815630728774 | 2.0602533011792543 | 0.14845657868001944 |
| XM_024168958.1-0/GRMZM2G032258_T01 | 0.1400493291742905 | NaN | NaN |
| augTM-XM_024164319.1-0/GRMZM2G177229_T01 | 0.17705564793043518 | 0.9271319586154255 | 0.19097135665008166 |
